# Selective abdominal venous congestion to investigate cardiorenal interactions in a rat model

**DOI:** 10.1371/journal.pone.0197687

**Published:** 2018-05-29

**Authors:** Jirka Cops, Wilfried Mullens, Frederik H. Verbrugge, Quirine Swennen, Carmen Reynders, Joris Penders, Jean-Michel Rigo, Dominique Hansen

**Affiliations:** 1 BIOMED–Biomedical Research Institute, REVAL–Rehabilitation Research Center, Faculty of Medicine and Life Sciences, Hasselt University, Diepenbeek, Belgium; 2 Doctoral School for Medicine and Life Sciences, Hasselt University, Diepenbeek, Belgium; 3 Department of Cardiology, Ziekenhuis Oost-limburg, Genk, Belgium; 4 Clinical Laboratory, Ziekenhuis Oost-Limburg, Genk, Belgium; 5 Heart Centre Hasselt, Jessa Hospital, Hasselt, Belgium; Max Delbruck Centrum fur Molekulare Medizin Berlin Buch, GERMANY

## Abstract

Abdominal congestion may play an important role in the cardiorenal syndrome and has been demonstrated to drive disease progression. An animal model for abdominal congestion, without other culprit mechanisms that are often present in patients such as low cardiac output or chronic kidney disease, might be interesting to allow a better study of the pathophysiology of the cardiorenal syndrome. The objective of this study was to develop a clinically relevant and valid rat model with abdominal venous congestion and *without* pre-existing heart and/or kidney dysfunction. To do so, a permanent surgical constriction (20 Gauge) of the thoracic inferior vena cava (IVC) was applied in male Sprague Dawley rats (IVCc, n = 7), which were compared to sham-operated rats (SHAM, n = 6). Twelve weeks after surgery, abdominal venous pressure (mean: 13.8 vs 4.9 mmHg, p < 0.01), plasma creatinine (p < 0.05), plasma cystatin c (p < 0.01), urinary albumin (p < 0.05), glomerular surface area (p < 0.01) and width of Bowman’s space (p < 0.05) of the IVCc group were significantly increased compared to the SHAM group for a comparable absolute body weight between groups (559 vs 530g, respectively, p = 0.73). Conventional cardiac echocardiographic and hemodynamic parameters did not differ significantly between both groups, indicating that cardiac function was not compromised by the surgery. In conclusion, we demonstrate that constriction of the thoracic IVC in adult rats is feasible and significantly increases the abdominal venous pressure to a clinically relevant level, thereby inducing abdominal venous congestion.

## Introduction

The heart and kidneys are closely related in heart failure and the term *cardiorenal syndrome* is used when acute or chronic dysfunction in one organ induces dysfunction in the other [1]. One third of decompensated heart failure patients demonstrates a worsening in renal function [2, 3]. Systemic venous congestion (backward failure) is an important culprit mechanism driving disease progression in heart failure patients with the cardiorenal syndrome [4, 5], as well as reductions in cardiac output (forward failure) [6]. Indeed, patients with acute decompensated heart failure who develop signs and symptoms of abdominal congestion, often experience worse outcomes [7–9]. Abdominal venous congestion is characterized by a strongly elevated intra-abdominal pressure (IAP) and central venous pressure (CVP). An increased IAP leads to compression of the kidneys and this pressure is transmitted to the thorax transdiaphragmatically, resulting in a diminished venous return to the heart [10, 11]. An increased CVP further leads to narrowing of the renal tubular lumen and an increased renal luminal pressure, resulting in a diminished transglomerular gradient and hence in a reduced glomerular filtration rate (GFR) [9, 12, 13].

The consequences of abdominal congestion are difficult to evaluate in patients, since concomitant forward failure or underlying chronic kidney disease very often coexist. As a result, efforts have been made to induce isolated renal venous congestion in animal models. In many of those experimental set-ups, the renal veins were selectively constricted in laboratory animals to induce congestion [14–17], whereas in patients, the abdomen’s entire venous system is congested, potentially explaining contradicting results in animal models versus clinical trials [18]. Establishing a well-characterized and easily accessible rat model that mimics abdominal venous congestion as seen in patients, is crucial to be able to better understand the role of abdominal venous congestion in the cardiorenal syndrome. This will allow us to examine the impact of different interventions in the treatment of the cardiorenal syndrome.

The goal of this study was to develop a rat model with abdominal venous congestion *without* pre-existing heart and/or kidney dysfunction, enabling to understand the impact of isolated venous abdominal congestion. We hypothesized that constriction of the thoracic inferior vena cava (IVC) would increase the abdominal venous pressure leading to abdominal venous congestion.

## Material and methods

### Animals and housing

This study conforms to the EU Directive 2010/63/EU for animal experiments and was approved by the Ethical Committee for Animal Experiments of Hasselt University, Belgium (protocol number: 201553). All efforts were made to minimize suffering. Animals were maintained in a temperature (22°C) and light (12:12h cycle) controlled animal facility. Rats were housed in pairs in Eurostandard type IV open-top cages enriched with wood shavings, nesting material and glass tunnels and had *ad libitum* access to a normal pellet diet and water, for the whole duration of the study. Animal health and behavior were monitored daily. During the first weeks, rats were handled daily to avoid handling-induced stress. At the end of the experiment or when humane endpoints were reached, animals were sacrificed by using an overdose of pentobarbital anesthesia (200 mg/kg, i.p.). Humane endpoints were defined as a decrease in bodyweight of more than 20% in the first week after surgery, or when the animal had difficulty breathing or was reluctant to move if stimulated, during three consecutive days after surgery.

### Study design

Twenty male healthy *Sprague-Dawley* rats (6 ± 1 weeks, 135 ± 15g, Charles River, France) were randomly divided into two groups: sham-operated rats (SHAM group, n = 6) were compared to inferior vena cava (IVC)-constricted rats (IVCc group, n = 7). Seven rats died as a result of the surgery to constrict the IVC to induce abdominal venous congestion, resulting in a mortality rate of 50% (7/14).

### Anesthesia

All experimental procedures such as surgery, obtaining blood samples, echocardiography and hemodynamic measurements, were performed under isoflurane using a mixture of isoflurane (1.5–2% volume supplemented with oxygen). Anesthesia was induced in an induction chamber (3% volume supplemented with oxygen) and continued by a face mask (1.5–2% volume supplemented with oxygen). After achieving a sufficient level of anesthesia, as indicated by the lack of a motoric response to a toe pinch, rats were put into a supine position onto a heating pad.

### Surgical procedure–constriction of IVC

**Fig 1:**
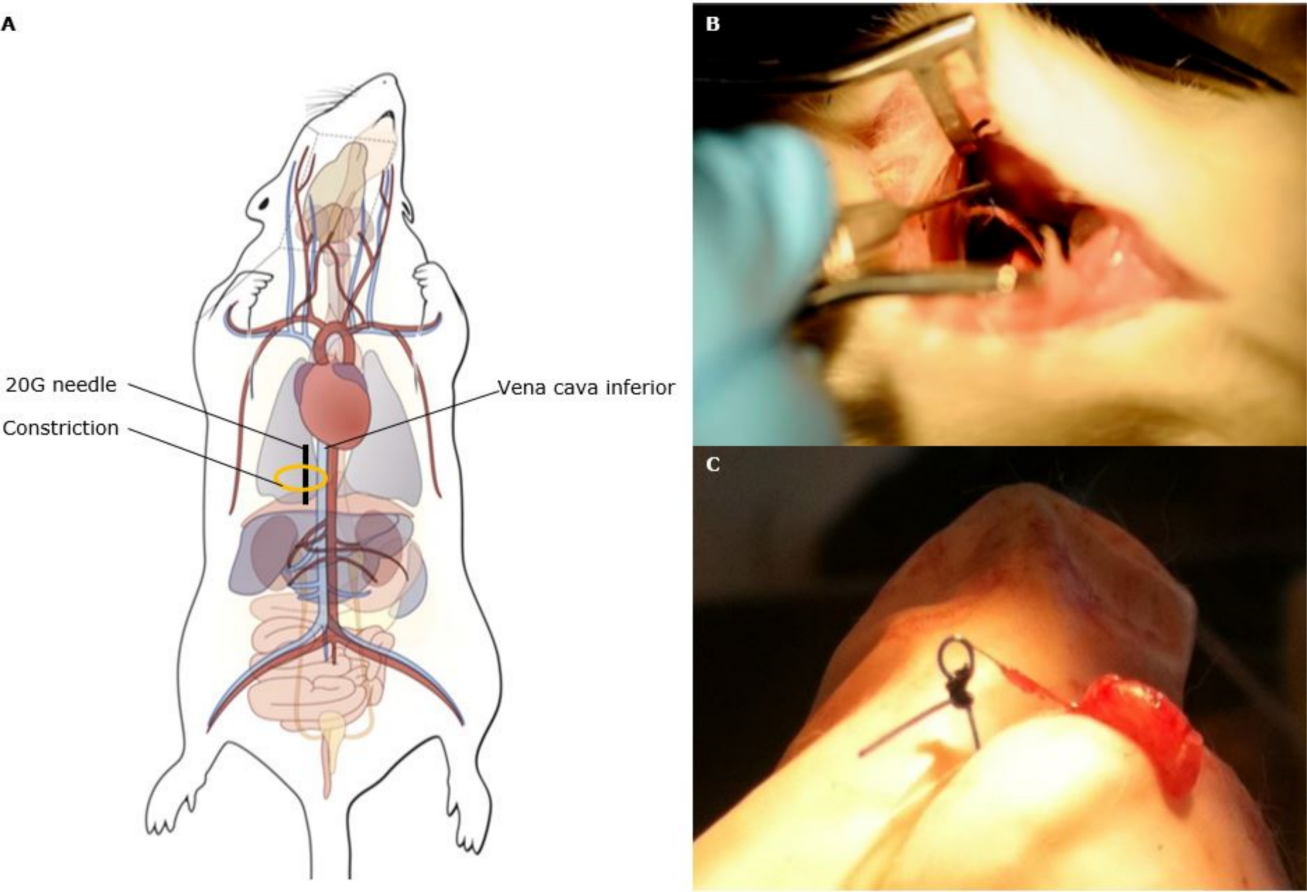
Characterization of a rat model of abdominal venous congestion by constriction of the thoracic inferior vena cava. Scheme of the experimental procedure to constrict the thoracic inferior vena cava (IVC) is shown in (A). Under isoflurane anesthesia and after endotracheal intubation, a right anterolateral thoracotomy was performed to reach the thoracic IVC and the vessel was dissected from the surrounding tissue. A permanent constriction was applied by tying a 6–0 prolene surgical wire (orange circle) around the thoracic IVC (light blue blood vessel) and a 20G needle (0.812 mm, black line), after which the needle was removed. Sham-operated rats were subjected to the same surgical procedure without application of the constriction. Figure adapted from Watts et al. 2012 [19]. Visualization of the constriction of the thoracic IVC *in situ* (B) and the constriction was correctly applied in all 7 IVCc rats, as confirmed by visualization of the constriction after killing the rats humanely (C).

After an adaptation period of one week, rats were anesthetized with isoflurane anesthesia in an induction chamber (3% volume supplemented with oxygen). Next, rats were transferred to a heated operating table and anesthesia isoflurane was maintained (1.5–2% volume supplemented with oxygen) using a face mask. After endotracheal intubation, rats were ventilated (1–1.5% volume supplemented with oxygen; Harvard apparatus, USA) and were fixed into a supine position onto the heated operating table. A right anterolateral thoracotomy between the fifth and sixth rib was performed. To reach the thoracic IVC, the pulmonary pleurae were punctured, the lungs were gently moved to the side and the IVC was dissected carefully from the surrounding tissue. A permanent constriction was applied by tying a surgical wire (6–0 prolene, VMD, Belgium) around the IVC and a 20 gauge (G, 0.812 mm) needle (Fig 1A and 1B), after which the 20G needle was removed and the wound was closed. As a result, the diameter of the IVC was reduced in a standardized manner to a known diameter of a 20G needle and importantly, no occlusion was performed. Sham-operated rats were subjected to the same surgical procedure without application of the constriction. After closing the wound, rats were turned on their right side and were extubated after restoration of spontaneous respiration. During a pilot experiment, we determined that the diameter of a 20G needle is the optimal diameter to increase the central venous pressure to a clinically relevant level (17.0 ± 2.6 mmHg, n = 5). When using a ≥ 21G needle, mortality rose to 100%.

### Recovery and post-operative analgesia and care

After surgery, rats were placed solely in clean cages until the next day, when they were placed back in their original cage. Meloxicam (1 mg/kg, Boehringer, Germany) was administered subcutaneously pre-operatively and was continued postoperatively twice a day for three consecutive days. Saline (0.9%, 0.01ml/g BW) was administered subcutaneously before extubation, to counteract dehydration. Antibiotics (10 mg/kg/day, Baytril, Bayer, Belgium) were administered via the drinking water to both groups for five consecutive days postoperatively.

### Blood and urine biochemical analysis

At baseline and 12 weeks after surgery, blood samples were obtained from the tail artery under isoflurane anesthesia (1.5–2% volume supplemented with oxygen). Blood samples were centrifuged (2000 rpm, 10 min) and plasma was preserved (-20°C) until later analysis. Plasma samples were analyzed for creatinine, cystatin C and urea using an automated analyzer (Cobas 8000 ISE module and Cobas 8000 c702 and c502 module, Roche diagnostics, Germany) [20–22].

At baseline and 12 weeks after surgery, twenty-four-hour urine samples were collected using standard rodent metabolic cages (technilab-BMI, the Netherlands) with glass play tunnels for cage enrichment. In the weeks before the actual 24-hour urine sample collection, rats were acclimated to the metabolic cages to avoid stress. Urine samples were centrifuged (1500 rpm, 5 min) and preserved (-20°C) until later analysis. Urine samples were analyzed for creatinine [23] and albumin using an automated analyzer (Cobas 8000 ISE module and Cobas 8000 c702 and c502 module, Roche diagnostics, Germany). Urinary kidney injury molecule 1 (KIM-1) concentrations were determined using the rat TIM-1/KIM-1/HAVCR DuoSet ELISA kit (DY3689, R&D Systems, USA) according to the manufacturer’s instructions and all measurements were performed in duplicate. Creatinine clearance was calculated from the following equation as previously described: creatinine clearance (ml/min/kg) = [(urinary creatinine x urinary volume)/(plasma creatinine x 1440 min) * 1000]/body weight [24].

### Echocardiography measurements

Echocardiography was performed at baseline and 12 weeks after surgery under isoflurane anesthesia in spontaneously breathing rats (1.5–2% volume supplemented with oxygen), using the GE VIVID *i* ultrasound machine and a 10S transducer (GE Vingmed Ultrasound, version 7.0.1, Norway). A standard parasternal long-axis image and a short-axis image at midventricular level were acquired using B-mode, at a temporal resolution of ≈ 200 frames per second. Left ventricular end-diastolic diameter (LVEDD), LV end-systolic diameter (LVESD), posterior and anterior wall thicknesses (PWT, AWT) were obtained from the parasternal short-axis view. Left ventricular end-diastolic volumes (EDV) and LV end-systolic volumes (ESV) were calculated as follows: π * D_M_^2^ * B/6. D_M_ indicates the systolic/diastolic diameter of the ventricle on midventricular short-axis view and B is the LV length on the parasternal long-axis image. Heart rate (HR) was determined by defining end systole and end diastole as the minimum and maximum LV short-axis area, respectively. Stroke volume (SV) was calculated as EDV–ESV. Cardiac output (CO) was calculated as SV * HR. Ejection fraction (EF) was calculated as [EDV-ESV]/EDV * 100 and expressed in %. Analysis was performed on an EchoPAC workstation (GE Vingmed Ultrasound, version 7.0.1, Norway).

### Hemodynamic measurements

Twelve weeks after surgery, invasive pressure measurements were performed under isoflurane anesthesia in spontaneously breathing rats (1.5–2% volume supplemented by oxygen). Briefly, a 2F micro tip high-fidelity pressure catheter (Millar Instruments, AD instruments, Germany), calibrated relative to atmospheric pressure before introduction, was first inserted into the right jugular vein and next into the left femoral vein and advanced into the abdominal IVC. After stabilization, the central venous pressure (CVP) was recorded. Finally, the catheter was advanced into the left ventricle (LV) via the right carotid artery. After stabilization of the animals, LV pressure (LVP) and its peak time derivatives (dP/dt_max_ and dP/dt_min_) were recorded. Left ventricular end-diastolic pressure (LVEDP) and the time constant of LV pressure decay during the isovolumic relaxation period (tau) were calculated using LabChart v7.3.7 software (Millar Instruments, AD instruments, Germany). Afterwards, rats were sacrificed with an overdose of pentobarbital (200 mg/kg, i.p.).

### Physical parameters

Rats were weighed at baseline and weekly during the 12-week follow-up period after surgery. At the end-of follow-up, after performing invasive hemodynamic measurements, rats were sacrificed and kidneys, liver, spleen and heart were excised for weighing and sampling. Tissues were fixed overnight in 4% paraformaldehyde and transferred to 70% ethanol until embedding in paraffin.

### Kidney injury

Kidney sections of five μm thick were stained using the Masson trichrome staining method. Sections were scanned using the Mirax Desk and observed at 20-times magnification using the Mirax viewer (Carl Zeiss MicroImaging, Germany). Fibrosis was assessed in four randomly chosen sections per rat, as previously described [25, 26]. The area of collagen deposition was outlined and quantified using an automated image analysis program (AxioVision 4.6, Carl Zeiss MicroImaging, Germany). Percentage fibrosis was calculated as the ratio of the area of collagen deposition to the global area.

### Kidney morphometry

Kidney morphometry was assessed in kidney sections stained using the Masson trichrome staining method. Glomerular surface area was measured in ten randomly chosen glomeruli per rat and width of Bowman’s space was measured five times per Bowman’s space in ten randomly chosen glomeruli per rat. Glomerular density was determined by calculating the number of glomeruli per total renal cortical area [27, 28]. For this purpose, five fields with a surface area of 3.14 mm^2^ were randomly selected and glomeruli were counted only considering well preserved structures [29]. All measurements were made using an analysis program (Pannoramic Viewer, 3DHISTECH, Hungary).

### Statistical analysis

Data are expressed as median [minimum; maximum]. After testing for normality using the Shapiro-Wilk normality test, parameters were compared using an unpaired t-test or a Mann-Whitney test or a two-way ANOVA when appropriate. Statistical analysis was performed using GraphPad Prism (GraphPad Prism Software 7.04, USA). A 2-tailed value of p < 0.05 was considered statistically significant.

## Results

### Mortality and post-operative complications

Of the original cohort of 20 rats, 7/14 animals survived the surgery to constrict the IVC and 6/6 rats the sham procedure, resulting in a mortality rate of 50% for the IVC constriction. The majority (n = 5) of deaths occurred during the recovery phase due to a low cardiac output syndrome as a result of the constriction, or due to a pneumothorax or tension pneumothorax. Two rats died of a bleeding when constriction was applied or due to a pulmonary bleeding. The constriction was correctly applied in all 7 IVCc rats, as confirmed by visualization of the constriction after killing the rats humanely (Fig 1C).

In the first two days after surgery, rats from both experimental groups suffered weight loss and demonstrated tachypnoea and hyperpnoea. In addition, the majority of rats displayed lethargy, a ruffled fur and porphyrin staining. However, from the third day onwards, these post-operative complications disappeared quickly.

### Physical parameters

Physical parameters of IVC-constricted and sham-operated rats are shown in Table 1. There was no difference in body weight (p = 0.73) or body weight gain (p = 0.72) 12 weeks after surgery. Spleen weight/tibia length ratio was significantly increased in the IVCc group (p < 0.01 respectively), compared to the SHAM group. Liver weight/tibia length ratio (p = 0.20), kidney weight/tibia length ratio (p = 0.78) and heart weight/tibia length ratio (p = 0.37) did not differ between groups (Table 1).

**Table 1:** Effect of twelve weeks of abdominal venous congestion on physical parameters.

Week 12	SHAM	IVCc	p-value
**Body weight (g) #**	524 [455;790]	540 [437;658]	0.73
**Body weight gain (g/12 weeks)**	344 [277;609]	371 [248;480]	0.72
**Liver weight/tibia length (mg/mm)**	388.5 [284.1;608.5]	485.4 [386.7;668.2]	0.20
**Spleen weight/tibia length (mg/mm)**	18.8 [17.1;22.9]	26.1 [22.5;31.8] **	0.003
**Kidney weight/tibia length (mg/mm)**	70.8 [65.2;93.6]	72.7 [64.4;90.5]	0.78
**Heart weight/tibia length (mg/mm)**	38.3 [36.4;43.5]	41.3 [33.3;54.8]	0.37

Data are shown as median [minimum; maximum] in sham-operated (SHAM, n = 6) and IVC-constricted rats (IVCc, n = 7). Data were analyzed using an unpaired t-test, when parametrically distributed according to the Shapiro-Wilk normality test. # denotes non-parametrically distributed data, which were analyzed using a Mann-Whitney test.

** denotes p < 0.01.

IVC = inferior vena cava, IVCc = IVC-constricted rats.

### Abdominal venous pressure

**Fig 2:**
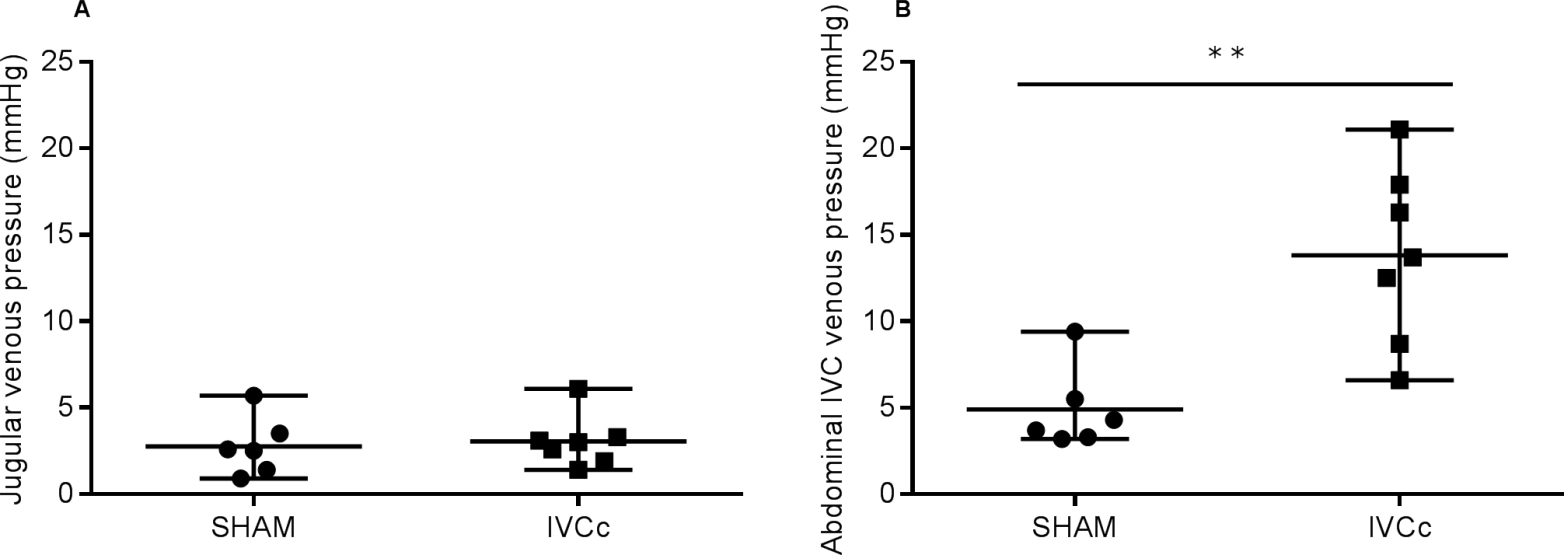
Constriction of the thoracic IVC induced an increase in abdominal central venous pressure below the constriction within 12 weeks after surgery. Venous pressure measured in (A) the jugular vein above the constriction level and (B) the abdominal IVC below the constriction level in sham-operated (SHAM, n = 6) and IVC-constricted rats (IVCc, n = 7). Based on the Shapiro-Wilk normality test, data were analyzed using an unpaired t-test (A) or a Mann-Whitney test (B). Data are shown as median, minimum and maximum. ** denotes p < 0.01. CVP = central venous pressure, IVC = inferior vena cava, IVCc = IVC-constricted rats.

To assess the development of abdominal venous congestion, venous pressure was measured above and below the constriction. Twelve weeks after surgery, the jugular venous pressure did not differ significantly between groups (2.6 [0.9;5.7] mmHg in the SHAM group versus 3.0 [1.4;6.1] mmHg in the IVCc group, p = 0.75). In contrast, abdominal venous pressure was significantly increased in IVC-constricted rats, compared to sham-operated rats (13.7 [6.6;21.1] mmHg versus 4.0 [33.2;9.4] mmHg, p < 0.01; Fig 2), which represents a 343% increase.

### Cardiac function

Baseline bodyweight and echocardiographic parameters were not different between SHAM and IVCc, except for a significantly increased left ventricular end-diastolic diameter (LVEDD) in IVCc rats (Table 2). Conventional echocardiographic (Table 2) and cardiac hemodynamic parameters (Table 3) did not differ significantly between groups 12 weeks after induction of abdominal venous congestion (p > 0.05).

**Table 2:** Conventional echocardiographic parameters at baseline and after twelve weeks of abdominal venous congestion.

	Baseline (week 0)		Week 12		p-value
	SHAM	IVCc	SHAM	IVCc	
**Body weight (g)**	180 [157;182]	170 [162;189]	524 [455;790]	540 [437;658]	0.72
**LVEDD (mm)**	4.5 [4.3;5.5]	5.3 [4.9;5.7]	7.7 [5.2;8.4]	8.6 [6.0;9.2]	0.99
**LVESD (mm)**	2.8 [1.8;3.2]	3 [2.6;3.4]	4.2 [2.4;5.7]	5.2 [3.8;5.6]	0.17
**PWT (mm)**	0.89 [0.61;1.04]	0.78 [0.62;0.95]	0.78 [0.64;0.98]	0.79 [0.62;0.92]	0.61
**AWT (mm)**	1.22 [0.98;1.38]	1.13 [1.00;1.55]	1.05 [0.91;1.17]	1.02 [0.81;1.26]	0.87
**HR (bpm)**	401 [372;413]	394 [359;462]	363 [330;417]	350 [305;467]	0.68
**EDV (μl)**^**#**^	101 [93;160]	137 [116;164]	444 [177;553]	493 [246;619]	0.42
**EDV/BW (μl/g)**^**#**^	0.56 [0.52;1.02]	0.76 [0.62;0.97]	0.86 [0.22;0.99]	1.02 [0.37;1.32]	0.73
**ESV (μl)**	27 [12;39]	29 [20;43]	95 [21;196]	129 [63;177]	0.30
**ESV/BW (μl/g)**	0.15 [0.07;0.22]	0.17 [0.12;0.23]	0.18 [0.03;0.35]	0.24 [0.14;0.34]	0.52
**CO (ml/min)**	31 [22;52]	44 [29;53]	119 [65;151]	116 [57;174]	0.79
**CO/BW (ml/min/g)**	0.17 [0.12;0.33]	0.27 [0.15;0.32]	0.22 [0.08;0.32]	0.22 [0.09;0.40]	0.56
**EF (%)**^**#**^	79 [59;90]	82 [63;87]	79 [65;88]	72 [49;77]	0.30

Data are shown as median [minimum; maximum] in sham-operated (SHAM, n = 6) and IVC-constricted rats (IVCc, n = 7). Data were analyzed using a two-way ANOVA, when parametrically distributed according to the Shapiro-Wilk normality test.

^#^ denotes not-parametrically distributed data, of which Δ change in time (week 12 –baseline) was analyzed using a Mann-Whitney test.

LVEDD = left ventricular end-diastolic diameter, LVESD = left-ventricular end-systolic diameter, PWT = posterior wall thickness, AWT = anterior wall thickness, HR = heart rate, EDV = end diastolic volume, ESV = end systolic volume, CO = cardiac output, EF = ejection fraction, BW = body weight, IVC = inferior vena cava, IVCc = IVC-constricted rats.

**Table 3:** Effect of twelve weeks of abdominal venous congestion on cardiac hemodynamic parameters.

Week 12	SHAM	IVCc	p-value
**MAP (mmHg)**	68.3 [65.4;71.1]	76.6 [63.8;84.1]	0.08
**LVP (mmHg)**	99.4 [93.9;111.9]	95.6 [78.3;101.3]	0.24
**LVEDP (mmHg)**	16.5 [8.4;27.4]	15.2 [7.6;36.4]	0.87
**dP/dt**_**max**_ **(mmHg/s)**	5976 [4689;6760]	5847 [4717;8346]	0.72
**dP/dt**_**min**_ **(mmHg/s)**	-5437 [-6065;-4334]	-5266 [-6482;-2724]	0.68
**Tau (s)**	0.011 [0.010;0.014]	0.012 [0.009;0.022]	0.54

Data are shown as median [minimum; maximum] in sham-operated (SHAM, n = 6) and IVC-constricted rats (IVCc, n = 7). Being parametrically distributed, data were analyzed using an unpaired t-test. MAP = mean arterial pressure, LVP = left ventricular pressure, LVEDP = left ventricular end-diastolic pressure, dP/dt_max_ = maximum value of the first derivate of LV pressure, dP/dt_min_ = minimum value of the first derivate of LV pressure, tau = time constant of LV pressure decay during the isovolumic relaxation period, IVC = inferior vena cava, IVCc = IVC-constricted rats.

### Renal function

**Fig 3:**
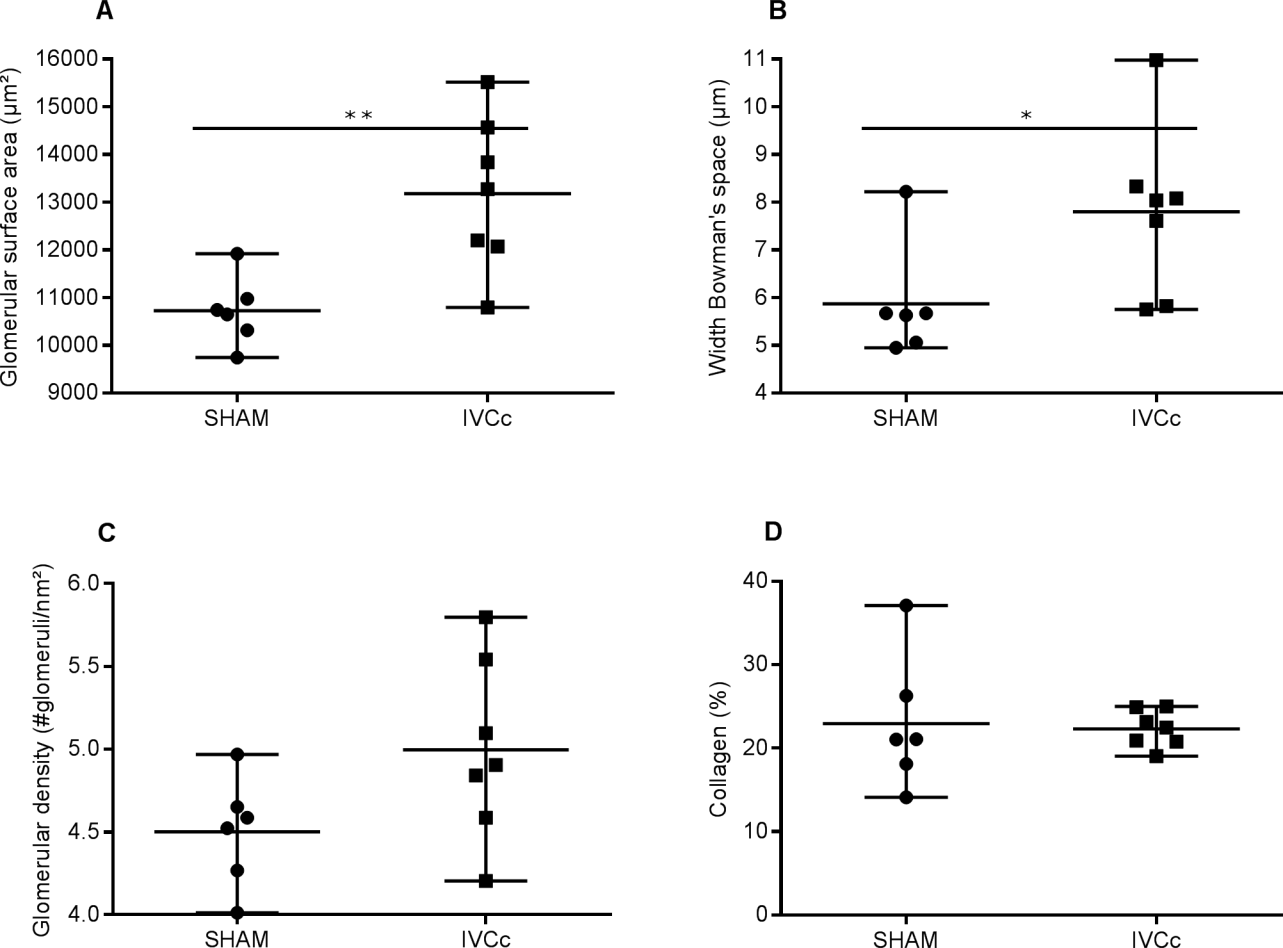
Abdominal venous congestion alters renal morphometry. Glomerular surface area (A), width of Bowman’s space (B), glomerular density (C) and quantification of total collagen from renal sections (D) of sham-operated (SHAM, n = 6) and IVC-constricted rats (IVCc, n = 7). Based on the Shapiro-Wilk normality test, data were analyzed using an unpaired t-test (A, C and D) or a Mann-Whitney test (B). Data are shown as median, minimum and maximum. * denotes p < 0.05, ** denotes p < 0.01. IVC = inferior vena cava, IVCc = IVC-constricted rats.

Baseline blood and urinary parameters were not different between SHAM and IVCc (S1 Table). Plasma creatinine and cystatin C and urinary microalbumin were significantly greater in IVCc versus SHAM rats, 12 weeks after surgery (p < 0.05, p < 0.01 and p < 0.05, respectively). Plasma urea (p = 0.66), twenty-four-hour urinary creatinine excretion (p = 0.82), urinary volume (p = 0.92), urinary KIM-1 (p = 0.11) and creatinine clearance (p = 0.14), were not statistically different between groups at 12 weeks after surgery (Table 4). Glomerular surface area and width of Bowman’s space were significantly greater in IVCc versus SHAM rats, 12 weeks after surgery (p < 0.01 and p < 0.05, respectively, Fig 3A and 3B). Glomerular density did not differ between groups (p = 0.08, Fig 3C). Renal collagen deposition, as indicated by the Masson trichrome staining method, did not differ between both groups at 12 weeks after surgery (p = 0.85, Fig 3D).

**Table 4:** Effect of twelve weeks of abdominal venous congestion on blood and urinary parameters.

Week 12	SHAM	IVCc	p-value
**Plasma creatinine (mg/dl)**	0.28 [0.22;0.33]	0.33 [0.30;0.40]*	0.03
**Plasma cystatin C (mg/dl)**	1.25 [0.59;1.75]	2.11 [1.72;2.68]**	0.002
**Plasma urea (mg/dl)**^**#**^	33 [31;37]	33 [28;41]	0.66
**Urinary creatinine excretion (mg/24h)**	94.0 [50.2;154.1]	101.0 [63.8;165.9]	0.82
**Urinary albumin (mg/g crea)**	24.8 [8.0;52.4]	86.4 [28.0;336.5]*	0.02
**Urine volume (ml/24h)**^**#**^	15.8 [6.5;23.0]	17.0 [7.5;24.0]	0.92
**Urinary KIM-1 (ng/g crea)**	515.4 [275.8;807.2]	665.2 [483.3;987.0]	0.11
**Creatine clearance (ml/min/kg)**	6.73 [6.26;8.61]	6.68 [4.59;7.05]	0.14

Data are shown as median [minimum; maximum] in sham-operated (SHAM, n = 6) and IVC-constricted rats (IVCc, n = 7). Data were analyzed using an unpaired t-test, when parametrically distributed according to the Shapiro-Wilk normality test.

^#^ denotes non-parametrically distributed data, which were analyzed using a Mann-Whitney test.

* denotes p < 0.05

** denotes p < 0.01.

KIM-1 = kidney injury molecule 1, IVC = inferior vena cava, IVCc = IVC-constricted rats.

## Discussion

By surgically narrowing the thoracic IVC, we were able to develop a rat model to selectively increase abdominal venous pressure without compromising cardiac function. This rat model offers the unique possibility of studying backward failure in heart failure and by extension in the cardiorenal syndrome, separately from forward failure. This is important since it remains unclear in which manner venous congestion contributes to cardiac and renal dysfunction in patients [7, 8].

The 20G diameter was chosen after a pilot experiment, indicating that this level of constriction is effective in increasing the abdominal venous pressure to a clinically relevant level. In addition, it was found that mortality increased when further decreasing the needle diameter. The average abdominal venous pressure of the IVCc group was significantly increased compared to sham-operated rats (p < 0.01). IVCc-constricted rats demonstrated a median abdominal venous pressure value of 13.7 [6.6;21.1] mmHg with values of 8 mmHg considered the upper limit of normal in patients [5]. We found no differences in cardiac hemodynamic parameters or conventional echocardiographic parameters twelve weeks after surgery. This was expected since a permanent constriction and no occlusion of the IVC is applied, probably inducing both superficial and deep venous collateralization [30]. Hence, preload remains sufficient to maintain cardiac function and to prevent cardiac unloading, as described in a canine model of thoracic IVC constriction [31–34]. In addition, these findings exclude the effects of a reduced cardiac output on organ functioning in our rat model, similar to the rat model of Simonetto et al. (2015) [35]. The significantly increased spleen weight/tibia length ratio is indicative of the involvement of the splanchnic system and is reported to be an indicator of portal hypertension [35, 36]. Based on these observations in the face of an unchanged body weight twelve weeks after surgery, it can be concluded that abdominal venous congestion is induced in IVC-constricted rats.

Twelve weeks after surgery, IVC-constricted rats demonstrated an augmented plasma creatinine and cystatin C level, indicating a worsened kidney function as a result of abdominal venous congestion. No significant difference in calculated renal creatinine clearance was observed between IVCc and SHAM group. Twenty-four-hour urinary creatinine excretion levels varied greatly between individual rats, probably due to evaporation of urine during the twenty-four-hour urine collection in the metabolic cages. In addition, tubular creatinine secretion increases in a setting of a worsening glomerular disease [37]. Urinary KIM-1 concentrations in the IVCc group tended to increase, although not reaching statistical significance (p = 0.11). KIM-1 is a transmembrane glycoprotein indicating proximal tubular injury [38] and is thought to detect congestion-induced renal injury more specifically than neutrophil gelatinase-associated lipocalin (NGAL) [39]. IVCc rats demonstrated albuminuria, suggesting a compromised integrity of the glomerular filtration barrier [40] and an altered kidney structure. Renal morphometry was altered in IVC-constricted rats, as shown by the significantly increased glomerular surface area and width of Bowman’s, indicating glomerulomegaly. Glomerular density was evaluated to determine if the observed glomerulomegaly was the result of a diminished number of glomeruli, leading to compensation of the remaining glomeruli to preserve the GFR. However, glomerular density did not differ between both groups. These results suggest a retrogradely conducted glomerular hypertension without a major impact on the GFR. Hypothetically, tubules are compressed due to the consequent renal congestion, leading to a raised luminal pressure and thereby attenuating the net pressure gradient across the glomerulus and decreasing the GFR [41]. Afferent vasodilation and efferent vasoconstriction may be initiated to increase the single nephron GFR [42], and in this way, an enlargement of the glomeruli is observed. To summarize, renal function and morphometry are affected by abdominal venous congestion, in contrast to an unaltered cardiac function. In this way, the role of abdominal venous congestion, as an important player in heart failure and the cardiorenal syndrome, is elucidated.

In the past, studies have been published describing constriction of the abdominal IVC in experimental animals. However, these studies did not meet the criteria of our rat model, since constriction was often applied on the abdominal IVC at the height of the renal veins or the liver [14, 43, 44]. Consequently, a local and no abdominal congestion was induced. Secondly, follow-up of these animals was short [45–47]–hours to days–and the constriction was sometimes removed [48, 49], hence the chronic impact of venous congestion could not be investigated in those studies. Moreover, hemodynamic measurements were not performed and it is not clear how the constriction was applied in these aforementioned experiments and therefore, it may be doubtful whether venous congestion was actually induced. In the last decades, a canine model of thoracic IVC constriction has been described, in which extensive neurohormonal activation and cardiac unloading is developed as a consequence of IVC constriction [31–34]. However, IVC constriction is removed in after seven to ten days and abdominal organ function is not investigated in these aforementioned studies. Because of these arguments, we opted to constrict the IVC in the thoracic cavity, hence above the diaphragm, in an easy-accessible rat model. In this way, all abdominal organs are affected and abdominal venous congestion is induced, similar to the clinical situation. The disadvantage of this method is that rats have to be intubated, which complicates the procedure, and mortality rises due to complications such as bleeding, pneumothorax and tension pneumothorax.

Pneumothorax and tension pneumothorax are important contributors to mortality. In the future, tension pneumothorax may be prevented by working around the lungs and by not moving the lungs. A pneumothorax may be prevented by establishing a vacuum in the thorax just before closing the wound, using a catheter and syringe and by sucking the air out of the thorax when the rat inhales. In general, the goal is to reduce the required time to perform the surgery, hence the rats are mechanically ventilated for a shorter period of time and will experience less adverse effects of isoflurane anesthesia.

In this study, only male rats were used to exclude effects from the female reproductive system. To investigate the effect of gender on the development of abdominal venous congestion, larger sample sizes are required. Since this was the first time such a rat model was developed, the validation of this rat model was the most important goal. In a next study, the influence of the female reproductive system can be investigated.

A possible limitation of this study is that isoflurane anesthesia was applied. Yang et al. (2014) showed that isoflurane decreased heart rate, blood pressure and myocardial contractility in rats at a high dose of 3% isoflurane. Isoflurane has a dose-dependent effect on cardiac hemodynamics, possibly elicited by dehydration and old age of the animals [50]. In contrast, Murakami et al. (2014) showed that inhalation anesthesia is suitable to conduct hemodynamic analyses in the rat [51]. Little is known about the effects of isoflurane on kidney function. According to Ruxanda et al. (2014), inhalation anesthesia such as isoflurane and sevoflurane, do not cause major renal histological changes except for a moderate vasodilatation at the corticomedullary junction [52]. In conclusion, determining the optimal isoflurane concentration and preconditioning is important. In this study, rats were preconditioned before hemodynamic measurements were performed and during these hemodynamic measurements, the most appropriate dose level of 1.5% isoflurane anesthesia was applied in both IVCc-constricted and sham-operated rats [53, 54], thereby excluding inter-group effects of isoflurane anesthesia. A second limitation of this study is the lack of evaluation of the GFR with an exogenous tracer, such as inulin, because creatinine is not only filtered by the glomeruli but is also secreted by proximal tubular cells. Inulin would be the ideal marker, since this polysaccharide is completely filtered by the glomeruli and is not secreted nor reabsorbed by the tubules [37]. Third, the magnitude of constriction cannot be determined, because the original IVC diameter may vary between rats and was not measured. In this study, the IVC was constricted against a 20G needle, afterwards resulting in the same remaining IVC diameter. However, when this surgical procedure is applied in animals with various bodyweight or ages, the magnitude of constriction and the coherent impact on hemodynamics, may differ from the results described in our study.

In conclusion, we demonstrated that permanent constriction of the thoracic IVC in adult rats significantly increases the abdominal venous pressure to a clinically relevant level, thereby inducing abdominal venous congestion. A rat model that mimics abdominal venous congestion (backward failure) *per se*, as seen in patients, is now available for further research to elucidate the pathogenesis of abdominal venous congestion, as an important contributor in the progression of the cardiorenal syndrome.

## Supporting information

S1 Table Baseline blood and urinary parameters. (DOCX)
