# Supplementary material for: Selective abdominal venous congestion to investigate cardiorenal interactions in a rat model
Source: PLoS One. 2018 May 29;13(5):e0197687. doi: 10.1371/journal.pone.0197687 (PMC5973578; doi:10.1371/journal.pone.0197687)
Supplement: S1 Table — (DOCX) [file pone.0197687.s001.docx]

**S1 Table: Table 1. Baseline blood and urinary parameters.**

| **Baseline (week 0)** | **SHAM** | **IVCc** | **p-value** |
| --- | --- | --- | --- |
| **Plasma creatinine (mg/dl)** | 0.15 [0.13;0.17] | 0.15 [0.12;0.17] | 0.79 |
| **Plasma cystatin C (mg/dl)** | 1.34 [0.64;2.04] | 1.92 [0.74;2.69] | 0.08 |
| **Plasma urea (mg/dl)^#^** | 26 [19;38] | 22 [20;29] | 0.42 |
| **Urinary creatinine excretion (mg/24h)** | 63.2 [35.6;105.3] | 57.9 [48.9;77.3] | 0.63 |
| **Urinary albumin (mg/g crea)** | 6.5 [1.3;12.1] | 6.4 [2.7;11.5] | 0.83 |
| **Urine volume (ml/24h)^#^** | 5.0 [4.0;10.0] | 6.3 [5.0;7.5] | 0.21 |
| **Urinary KIM-1 (ng/g crea)** | 1618.0 [1290.0;2245.0] | 2008 [1653;2284.0] | 0.07 |
| **Creatine clearance (ml/min/kg)** | 9.85 [6.34;12.16] | 11.61 [10.58;12.49] | 0.05 |

Data are shown as median [minimum; maximum] in sham-operated (SHAM, n = 6) and IVC-constricted rats (IVCc, n = 7). Data were analyzed using an unpaired t-test, when parametrically distributed according to the Shapiro-Wilk normality test. ^#^ denotes non-parametrically distributed data, which were analyzed using a Mann-Whitney test. KIM-1 = kidney injury molecule 1, IVC = inferior vena cava, IVCc = IVC-constricted rats.
